# Are everyday sadists specifically attracted to violent video games and do they emotionally benefit from playing those games?

**DOI:** 10.1002/ab.21810

**Published:** 2018-12-26

**Authors:** Tobias Greitemeyer, Niklas Weiß, Tobias Heuberger

**Affiliations:** ^1^ Institut für Psychologie Universität Innsbruck Innsbruck Austria

**Keywords:** aggression, Dark Triad, everyday sadism, video games

## Abstract

The present research tested the hypothesis that everyday sadists show a distinct preference for violent video games and examined the relationship between everyday sadism and participant's mood after violent video game play. In Study 1, participants watched three trailers for video games that differed in their level of violent content. Whereas everyday sadists were attracted to a violent video game, there was no significant positive association between everyday sadism and attraction to the nonviolent video games. Study 2 showed that after playing a violent video game, there was a significant positive relationship between everyday sadism and participant's positive mood and a negative relationship between everyday sadism and participant's negative mood. In contrast, after playing a nonviolent video game, the relationship between everyday sadism and participant's negative mood was less pronounced. Overall, these studies show that everyday sadists specifically like to play violent video games and suggest that this tendency is adaptive in that they emotionally benefit from playing violent video games.

## 1 INTRODUCTION

Researchers have been interested in the relationship between the Big 5 personality dimensions and the preference for playing violent video games. For example, in one study (Anderson et al., 2004), amount of violent video game play was negatively associated with agreeableness and conscientiousness. In another study (Chory & Goodboy, 2011), participants lower in agreeableness and higher in openness were more likely to play violent video games. In the present research, we focus on the relationship between dark personality traits (in particular, everyday sadism) and (a) attraction to violent video games and (b) the emotional consequences of playing a violent video game.

Recent theorizing suggests that there are four dimensions that characterize the dark side of human personality (called the Dark Tetrad): narcissism, Machiavellianism, psychopathy, and everyday sadism (Chabrol, Van Leeuwen, Rodgers, & Séjourné, 2009; Paulhus, 2014). In brief, narcissists have a grandiose sense of self‐importance and superiority, Machiavellians manipulate and exploit others, psychopaths are characterized by callousness, thrill seeking, and unemotionality, and everyday sadists obtain pleasure from cruel acts of behavior. Of these dimensions, it appears that everyday sadism has the most robust association with the extent to which individuals play violent video games (Greitemeyer, 2015).

Everyday sadism is positively related to the other dark personalities, in particular psychopathy and Machiavellianism (e.g., Book et al., 2016), but is conceptually distinct in that this trait particularly predicts experiencing pleasure from causing harm. For example, individuals who score high on the personality trait of everyday sadism find more pleasure in killing bugs and harming unknown persons than do nonsadists. They are even willing to spend considerable time on boring tasks to gain the opportunity to hurt innocent victims (Buckels, Jones, & Paulhus, 2013). Given that most modern violent video games involve seriously harming other game characters, they may provide the opportunity for everyday sadists to satisfy their need for cruelty. In line with this reasoning, research has shown that everyday sadists are attracted to violent video games (Gonzalez & Greitemeyer, 2018; Greitemeyer, 2015). Importantly, this relationship held when controlling for the influence of trait aggression and the Dark Triad (narcissism, Machiavellianism, and psychopathy), suggesting that everyday sadists in particular are attracted to violent video games. A longitudinal investigation (Greitemeyer & Sagioglou, 2017) showed that everyday sadism at Time 1 significantly predicted the amount of violent video game play at Time 2 even when controlling for the influence of amount of violent video game play at Time 1. It thus appears that the relationship between everyday sadism and the amount of playing violent video games is indeed in part due to everyday sadists being more attracted to violent video game play (although the relationship is bidirectional: violent video game play also increased sadistic tendencies).

These findings strongly suggest that everyday sadists are more likely than others to seek out violent video contents. What is unclear, however, is whether everyday sadists are not only attracted to violent video games but to nonviolent video games as well. It may simply be that everyday sadists like playing video games, so the positive relationship between everyday sadism and the amount of violent video game play could be a byproduct of everyday sadists’ tendency to play any kind of video game and may speak against the hypothesis that everyday sadists are specifically attracted to violent video games. Hence, in Study 1 of the present research, we examined the relationship between everyday sadism and attraction to three video games that differ in whether they contain violent content. If our reasoning is correct, everyday sadists will be attracted to violent video games but they will not be attracted to nonviolent video games.

Study 2 addressed the emotional consequences of playing video games. Research suggests that individuals often seek out video games because of the expectation that video game play reduces stress (Whitbourne, Ellenberg, & Akimoto, 2013) and regulates the player's emotions (Olson, 2010). Some studies (e.g., Bowman & Tamborini, 2015; Russoniello, O'Brien, & Parks, 2009) do find that playing preferred video games has a positive impact on the player's mood. Given that everyday sadists seem to be strongly inclined to play violent video games, we expected them to emotionally benefit from playing such a game.

To rule out the possibility that playing any type of video game improves everyday sadists’ mood, we also examined the impact of playing a nonviolent video game on the player's mood change. As noted above, we reasoned that everyday sadists are attracted to violent video games because these games can be employed to satisfy their needs for cruelty. In contrast, playing a nonviolent video game does not serve this goal and thus everyday sadists should not be attracted to nonviolent video games. Given that only playing preferred video games improves the player's mood (Bowman & Tamborini, 2015; Russoniello et al., 2009), everyday sadists should not emotionally benefit from playing a nonviolent video game.

Our university requires no formal approval from an ethics committee if the research is in accordance with guidelines of the German Psychological Society. In both studies, only participants that were older than 18 years were allowed to participate. It was also stressed that the data are analyzed anonymously and that participation in the study is voluntary. Due to time constraints (the present studies were part of students’ projects), we did not determine sample sizes by doing a priori power analyses but rather tried to run as many participants as possible during one semester. A sensitivity analysis showed that the current samples (*N*s = 243 & 216) provided 80% power to detect an effect of *r* = 0.18 (0.19, respectively). Given that the typical published effect size in social psychology is about *r* = 0.21 (Richard, Bond, & Stokes‐Zoota, 2003), statistical power should be sufficient. In both studies, no attention checks were employed and participants received no explicit information about the respective aim of the study. No suspicion checks were employed in either study.

## 2 STUDY 1

The aim of Study 1 was to examine the notion that individuals who score relatively high in everyday sadism show a distinct preference for violent video games. To this end, participants watched trailers for three video games. Liking of and interest in (watching time) each video game was assessed. One of the video games contained explicit violence. It was predicted that everyday sadism would be positively associated with liking, and interest in, this video game. We further examined whether these relationships remained significant when controlling for the influence of trait aggression and the Dark Triad. Such a destructive testing approach (Prot & Anderson, 2013) would provide strong evidence for the proposed link between everyday sadism and the preference for violent video games if the inclusion of these competitor variables fails to break the relationship. The second video game contained no violence (neutral video game), whereas the third video game was a nonviolent shooter game. Given that nonviolent games do not provide an opportunity to satisfy the need for cruelty, everyday sadists should not be attracted to these video games. Hence, the relationships between everyday sadism and liking of and interest in these video games, respectively, should not be significantly positive. Moreover, the relationships between everyday sadism and liking of and interest in the violent video game, respectively, should be more pronounced than the relationships between everyday sadism and liking of and interest in the two other video games.

### 2.1 Method

#### 2.1.1 Participants, procedure, and materials

The link to an online questionnaire was distributed via social networks. Participants were also invited via a university mailing list. One participant did not complete the everyday sadism scale and was thus excluded from the analyses. The final sample comprised 242 individuals who provided data for all relevant variables (87 females, 155 males; mean age = 24.9 years, SD = 5.7). There was no compensation for participation.

After providing demographics, the Dark Triad was assessed. To this end, the Short Dark Triad by Jones and Paulhus (2014) was employed. All subscales contain nine items. Sample items: “People see me as a natural leader” (narcissism, α = .68), “I like to use clever manipulation to get my way.” (Machiavellianism, α = .75), and “People who mess with me always regret it.” (psychopathy, α = .69). Everyday sadism was assessed with the expanded version of the Comprehensive Assessment of Sadistic Tendencies (CAST, Buckels, Trapnell, & Paulhus, 2014). The scale contains 11 items (α = .80). Sample item: “I enjoy physically hurting people.”^1^ To measure trait aggression, participants responded to the short version of the Buss and Perry aggression questionnaire (Bryant & Smith, 2001). The scale contains 12 items (α = .80). Sample item: “Given enough provocation, I may hit another person.” All items were assessed on a scale from 1 to 5.

Afterwards, participants watched three video game trailers. After each trailer, liking of the video game was assessed by three items (e.g., How much would you like to play this game?”). These items were assessed on a scale from 1 to 4. For each trailer, these three items were highly correlated and were thus collapsed into one liking scale (all αs >.87). Participants had to watch each trailer for at least ten seconds before they could proceed. They were free to watch the entire trailer and the time participants watched each trailer was assessed. This measure was employed as a proxy for interest in the video game. The first trailer was for Splatoon, which is a third‐person nonviolent shooter game. Rather than shooting missiles, the player uses ink (or colors). The Entertainment Software Self‐Regulation (USK) organization approves the game for children aged six and above. The second trailer was for Sims 4, which is a life simulation game (neutral video game). The USK approves the game for children aged 12 and above. The third trailer was for Mortal Kombat X, which is a fighting game (violent video game). The USK does not approve the game for anyone under 18. All participants watched the three trailers in the same order.

### 2.2 Results

Descriptive statistics and intercorrelations of all measures are reported in Table 1. As can be seen, everyday sadism was positively associated with liking of the violent video game. In contrast, everyday sadism was not significantly associated with liking the nonviolent shooter game and was even negatively associated with liking of the neutral video game. A similar pattern of findings occurred for trait aggression and the Dark Triad, although the correlations were smaller. To examine whether everyday sadism is associated with liking the violent video game when controlling for the impact of trait aggression and the Dark Triad, a simultaneous regression was performed on the data. Everyday sadism, trait aggression, and the Dark Triad were used as predictors for liking the violent video game. In addition, bootstrapping analysis based on 1,000 bootstrap samples was run. The overall regression was significant, *F*(5, 241) = 7.96, *p* < .001. Most importantly, everyday sadism was still significantly associated with liking the violent video game, β = .19, *p* = .020 (bootstrapping coefficient = 0.28, 95%CI [0.01, 0.56]). None of the other predictors received a significant regression weight (trait aggression: β = .06, *p* = .427, bootstrapping coefficient = 0.08, 95%CI [−0.14, 0.31]; narcissism: β = .03, *p* = .705, bootstrapping coefficient = 0.04, 95%CI [−0.19, 0.26]; Machiavellianism: β = .03, *p* = .660, bootstrapping coefficient = 0.04, 95%CI [−0.14, 0.23]; psychopathy: β = .24, *p* = .055, bootstrapping coefficient = 0.24, 95%CI [−0.07, 0.49]).

**Table 1:** Means, standard deviations, and bivariate correlations (study 1)

	*M*	*SD*	1	2	3	4	5	6	7	8	9	10
1. Everyday sadism	1.77	0.61										
2. Trait aggression	2.21	0.66	.44***									
3. Narcissism	2.78	0.60	.45***	.14*								
4. Machiavellianism	3.25	0.66	.25***	.22**	.24***							
5. Psychopathy	2.16	0.61	.62***	.52***	.37***	.38***						
6. Liking of nonviolent	2.34	0.87	.02	−.03	−.14*	−.07	−.04					
7. Liking of neutral	2.25	0.87	−.14*	−.06	−.01	−.04	−.08	.12				
8. Liking of violent	1.87	0.89	.34***	.24***	.19**	.16*	.34***	.20**	−.04			
9. Interest in nonviolent	61	33	−.04	−.04	−.07	.00	−.04	.15*	−.01	−.13		
10. Interest in neutral	81	36	−.17**	−.02	−.07	.00	−.06	.01	.04	−.18**	.56***	
11. Interest in violent	63	47	.15*	.12	.17**	.11	.16*	.09	−.03	.08	.49***	.38***

*p < .05, **p < .01, ***p < .001.

Similarly, everyday sadism was positively associated with interest in the violent video game, whereas everyday sadism was not associated with interest in the nonviolent shooter game and was negatively associated with interest in the neutral video game. As before, the pattern of findings for trait aggression and the Dark Triad was similar, but less pronounced. However, everyday sadism did not significantly predict interest in the violent video game in the regression (all predictors were non‐significant).

Fisher's *z* tests showed that the correlation between everyday sadism and liking of the violent video game was significantly different from the correlations between everyday sadism and liking the nonviolent shooter game, *z* = 4.45, *p* < .001, and between everyday sadism and liking the neutral video game, *z* = 5.75, *p* < .001, respectively. Moreover, the correlation between everyday sadism and interest in the violent video game was significantly different from the correlations between everyday sadism and interest in the nonviolent shooter game, *z* = 2.92, *p* < .001, and between everyday sadism and interest in the neutral video game, *z* = 4.48, *p* < .001, respectively.

### 2.3 Discussion

Study 1 supports the hypothesis that everyday sadists show a distinct preference for playing video games where they can cause virtual harm. As expected, everyday sadism was positively associated with liking a violent video game and this relationship held when controlling for trait aggression and the Dark Triad. Most of these findings were corroborated with the behavioral measure of interest in the violent video game (with the exception that the influence of everyday sadism on interest in the violent video game did not hold when controlling for trait aggression and the Dark Triad).

Everyday sadists were attracted to the violent video game, but they were not attracted to the nonviolent video games. The relationships between everyday sadism and liking of and interest in the two nonviolent video games were not significantly positive, and were less pronounced than the relationships between everyday sadism and liking, and interest in, the violent video game. There was even a negative relationship between everyday sadism and liking, and interest in, the neutral video game. Overall, Study 1 shows that everyday sadists are attracted to violent video games but that they are not attracted to video games that do not contain violence.

## 3 STUDY 2

Study 2 examined the impact of video game play on mood change. Study 1 indicated that everyday sadists show a distinct preference for violent video games. It was reasoned that because playing preferred video games improves the player's mood (Bowman & Tamborini, 2015; Russoniello et al., 2009), there should be a significant relationship between everyday sadism and participant's mood after playing a violent video game. In contrast, no significant relationship was expected after nonviolent video game play. To test these ideas, participants’ mood was assessed twice, once before they played a video game and once after video game play and the partial correlation between everyday sadism and participant's post‐game mood was calculated (controlling for participant's pre‐game mood). To allow causal conclusions, participants were randomly assigned to either play a violent or a nonviolent video game.

As secondary goals, Study 2 examined whether everyday sadists would be more likely than others to play violent video games, which is a replication of previous work (Greitemeyer, 2015). Moreover, during violent video game play, the number of times the player kills other game characters was recorded by the video game. Given that everyday sadists enjoy causing harm more than others, we predicted a positive relationship between everyday sadism and number of kills.

### 3.1 Method

#### 3.1.1 Participants, procedure, and materials

Two‐hundred and sixteen students from an Austrian university participated (96 females, 118 males, 2 others; mean age = 22.9 years, SD = 4.0). Participants either received course credit or 5 Euros (approximately 6 U.S. dollars).

After providing demographics, participants completed the 18 items from the CAST (α = .82). We then assessed participant's amount of violent video game play. For each of their three favorite video games, participants indicated how often they play the game and rated how violent the content of the game is. The scale for both items was from 1 to 7. For each video game, frequency of game play was multiplied by violent content (e.g., Anderson & Dill, 2000). These three violent video game exposure scores were then summed. If participants did not play video games, they received a score of zero.

Afterwards, we employed the PANAS (Watson, Clark, & Tellegen, 1988) to assess pre‐game positive (α = .87) and negative mood (α = .84). Participants indicated to what extent they felt twenty different emotions at the moment. There were ten items measuring positive mood (e.g., “happy”) and ten items measuring negative mood (e.g., “sad”). The scale was from 1 to 5. We added one item that assessed participant's general mood. The scale for this item was from −5 (*very bad*) to +5 (*very good*).

All participants then played the video game Grand Theft Auto: San Andreas (GTA) for ten minutes. In the violent condition, participants had to defend themselves against attacking police men. To minimize frustration and to limit the impact of previous game experience, the game was modified so that participants had unlimited access to weapons and ammunition and could not be killed. However, they could be arrested by the police. If they got arrested, the game had to be restarted. For those participants that were not arrested, the number of kills was recorded (*N* = 49). (This parameter is not meaningful for participants that got arrested because with the restart the number of kills was reset to zero.) In the nonviolent condition, participants took part in a triathlon and competed against computer opponents. To ensure that participants did not behave aggressively during video game play and in line with previous work showing that punished violent behavior within a video game reduces violent in‐game behavior (Carnagey & Anderson, 2005), participants learned that the use of violence led to immediate disqualification (and a new game start).

After playing the video game, post‐game mood was assessed, using the same items (positive mood: α = .90; negative mood: α = .87). Participants then indicated how violent the video game was. This item was employed as a manipulation check. They also responded to four questions assessing liking of the video game (e.g., “How much did you enjoy playing the video game?,” α = .84). All video game ratings were assessed using a scale from 1 (*not at all*) to 5 (*very much*). To assess participant's previous GTA experience, they reported how often they played GTA before, using a scale from 0 (*never*) to 3 (*more than 50 times*). Because the study was part of a student project, additional measures were employed. For example, we examined whether everyday sadists were more likely to believe than others that violent video games do not cause aggression (they were). Because these measures are not relevant for the present purposes, we do not report them.

### 3.2 Results

The manipulation check was successful, *t*(214) = 23.77, *p* < .001, *d* = 3.32. The content of the violent video game (*M* = 4.01, *SD* = 1.05) was perceived as being more violent than the content of the nonviolent video game (*M* = 1.22, *SD* = 0.56). Liking the two video games was relatively similar (violent video game: *M* = 3.06, *SD* = 0.97, nonviolent video game: *M* = 2.84, *SD* = 0.99), *t*(214) = 1.69, *p* = .093, *d* = 0.22. Moreover, everyday sadism was positively associated with amount of violent video game play, *r*(216) = .33, *p* < .001.

Our main hypothesis was that after playing the violent video game, everyday sadism should be related to participant's mood. Because the PANAS mood measures were not significantly correlated (see Table 2), partial correlation coefficients for the relationship between everyday sadism and participant's post‐game mood (controlling for participant's pre‐game mood) were separately calculated for each of the three mood measures. As predicted, in the violent video game condition, the partial correlations between everyday sadism and positive mood, *r*(113) = .36, *p* < .001, and general mood, *r*(113) = .30, *p* = .001, respectively, were significantly positive, whereas the partial correlation between everyday sadism and negative mood was significantly negative, *r*(113) = −.27, *p* = .003. Unexpectedly, in the nonviolent video game condition, the partial correlation between everyday sadism and positive mood was also significant, *r*(97) = .20, *p* = .045. In contrast, the partial correlations between everyday sadism and general mood, *r*(97) = .18, *p* = .081, and negative mood, *r*(97) = .06, *p* = .535, respectively, were non‐significant.

**Table 2:** Means, standard deviations, and bivariate correlations (study 2)

	*M*	SD	1	2	3	4	5	6
1. Everyday sadism	2.15	0.69						
2. Positive mood Time 1	3.08	0.66	.21**					
3. Negative mood Time 1	1.37	0.44	.20**	−.01				
4. General mood Time 1	2.46	1.56	.01	.53***	−.37***			
5. Positive mood Time 2	3.10	0.79	.35***	.58***	.05	.33***		
6. Negative mood Time 2	1.48	0.58	−.00	.11	.53***	−.08	−.04	
7. General mood Time 2	2.58	1.61	.20**	.32***	−.22**	.62***	.57***	−.30***

**p < .01, ***p < .001.

We then examined whether the partial correlations between everyday sadism and post‐game mood differed in the two video game conditions. In fact, a Fisher's *z* test showed that the partial correlation between everyday sadism and negative mood significantly differed between the violent and the nonviolent condition, *z* = 2.34, *p* < .001. In contrast, for both positive, *z* = 1.24, *p* = .108, and general mood, *z* = 0.91, *p* = .182, the Fisher's *z* test was non‐significant.

Next, we tested whether everyday sadism would be associated with number of kills during game play. The correlation was significantly positive, *r*(49) = .47, *p* = .001. However, killing game characters may not only depend on the players’ motivation but also on their ability. Indeed, previous GTA experience was significantly associated with number of kills, *r*(49) = .45, *p* = .001. Hence, we tested whether the relationship between everyday sadism and number of kills would hold when controlling for previous GTA experience. In a multiple regression, everyday sadism and previous GTA experience were used as predictors of the number of kills. The overall regression was significant, *F*(2, 46) = 10.40, *p* < .001. Everyday sadism was still significantly associated with number of kills, β = .36, *p* = .009 (bootstrapping coefficient = 19.64, 95%CI [6.74, 45.27]. Previous GTA experience was also a significant predictor, *β* = .32, *p* = .020 (bootstrapping coefficient = 11.83, 95%CI [3.35, 21.35]).

### 3.3 Discussion

Study 2 showed that everyday sadists’ attraction to violent video games has some rational roots in that violent video game play has a positive impact on everyday sadists’ mood. As predicted, after playing a violent video game, there was a positive relationship between everyday sadism and participant's positive mood and a negative relationship between everyday sadism and participant's negative mood. However, after nonviolent video game play, there was also a positive relationship between everyday sadism and participant's positive mood. Moreover, the correlation coefficients between everyday sadism and participant's positive and general mood did not significantly differ between the two video game conditions. In contrast, after nonviolent video game play, the relationship between everyday sadism and participant's negative mood was non‐significant, and the correlation coefficient between everyday sadism and participant's negative mood did differ between the two video game conditions. It thus appears that violent more than nonviolent video game play is associated with decreases in everyday sadists’ negative mood, whereas positive affective states are less distinctly affected.

Study 2 also provides a conceptual replication of Study 1 by showing that everyday sadists seem to be attracted to violent video games. As in previous research (Greitemeyer, 2015), there was a positive relationship between everyday sadism and the reported amount of violent video game play. Study 2 thus reveals that everyday sadists are more likely to play violent video games than others and that this tendency may have a positive impact on their mood. Study 2 also showed that everyday sadists committed more killings during violent video game play than did nonsadists. Importantly, this relationship remained significant when controlling for previous game experience, suggesting that everyday sadists kill more game characters not only because of their greater ability but also because of their motivation. This finding provides suggestive evidence that everyday sadists indeed use violent video games to satisfy their need for cruelty. However, we acknowledge that due to the relatively small number of participants that could be used for these analyses, future work is needed before strong conclusions are warranted.

## 4 GENERAL DISCUSSION

The present studies had two main goals. First, previous research has shown that everyday sadists are attracted to violent video games (Greitemeyer, 2015; Greitemeyer & Sagioglou, 2017), but it is not known whether everyday sadists also like to play video games that do not involve causing virtual harm. As everyday sadists should be particularly inclined to play video games that satisfy their needs for cruelty, we expected a specific link between everyday sadism and the preference of violent video games. Supporting our hypothesis, Study 1 showed that there was a strong relationship between everyday sadism and liking, and interest in, a violent video game and these relationships were more pronounced than the relationships between everyday sadism and liking and interest in nonviolent video games. The present research thus demonstrates that everyday sadists have a clear preference for committing virtual harm during video game play over playing video games that do not contain violent action.

In fact, everyday sadism was not significantly associated with liking of and interest in the nonviolent shooter game and it was even negatively associated with liking of and interest in the neutral video game. Future research may examine whether everyday sadists are particularly disinclined to play certain kinds of video games. Given that everyday sadists enjoy causing harm, they may be less willing than others to benefit others. Many video games include the opportunity to behave prosocially, with the typical finding that these video games increase helping behavior (Gentile et al., 2009; Greitemeyer & Cox, 2013; Greitemeyer & Osswald, 2010; Prot et al., 2014). In a nationally representative survey (Lenhart et al., 2008), 78% of the respondents reported helping other game characters and 76% played video games cooperatively with others. Whereas these prosocial and cooperative video games seem to be attractive to many, we would expect that everyday sadists display a dislike of those games.

When we refer to participants as being everyday sadists, it is important to keep in mind that the overall mean level of everyday sadism in both studies was considerably below the midpoint of the scale. That is, in our samples, participants who showed a preference for violent video games did not have high scores on everyday sadism; they just had higher scores than participants who were less favorable toward violent video games.

It is noteworthy that the relationship between everyday sadism and the preference for violent video games remained significant when controlling for the impact of trait aggression and the Dark Triad, suggesting that everyday sadists’ preference for violent video games is *not* due to them being more aggressive and scoring high on narcissism, Machiavellianism, and psychopathy. This finding provides further evidence that everyday sadism is conceptually and empirically distinct from other forms of the dark side of human personality (see also Buckels et al., 2014; Pfattheicher & Schindler, 2015; Plouffe, Saklofske, & Smith, 2017).

Our second goal was to address whether everyday sadists’ preference for violent video games would be adaptive in that playing those games is associated with increases in positive affect and decreases in negative affect. Previous research (Bowman & Tamborini, 2015; Russoniello et al., 2009) has shown that playing preferred video games improves the player's mood. Given that Study 1 showed that everyday sadists are attracted to violent video games but not to nonviolent video games, we predicted that everyday sadists would emotionally benefit from playing a violent video game but would not benefit from playing a nonviolent video game. These hypotheses received partial support. After playing a violent video game, there were indeed significant relationships between everyday sadism and our mood measures. However, contrary to our expectations, after nonviolent video game play, there was also a positive relationship between everyday sadism and participant's positive mood. In contrast, after nonviolent video game play, the relationship between everyday sadism and participant's negative mood was not reliable and the correlation coefficient between everyday sadism and participant's negative mood was more pronounced in the violent video game condition. The expectation that aggression improves affective states has been shown to be associated with aggressive action (Bushman, Baumeister, & Phillips, 2001; Chester & DeWall, 2017). Likewise, everyday sadists may use violent video games to repair their mood. However, whether everyday sadists indeed actively seek out violent video games in order to regulate their mood was not tested in the present research and is thus an interesting avenue for future research.

To conclude, the present studies provide evidence that everyday sadists are attracted to video games that offer an opportunity to satisfy their need for cruelty, whereas they are not attracted to video games where no virtual harm can be committed. These preferences seem to be adaptive in that everyday sadists emotionally benefit from playing violent video games. Playing violent games has been shown to increase aggression and thus has negative interpersonal consequences (Anderson et al., 2010; Greitemeyer & Mügge, 2014). On the other hand, at least for everyday sadists, the intrapersonal consequences seem to be positive.

## ENDNOTE

1 The CAST includes 18 items in total. Seven items that assess vicarious sadism were not included in the analyses, because they are conceptually close to the main dependent measures (attraction to violent video games). However, including these items does not change the pattern of findings.
